# Time to Culture Conversion and Regimen Composition in Multidrug-Resistant Tuberculosis Treatment

**DOI:** 10.1371/journal.pone.0108035

**Published:** 2014-09-19

**Authors:** Dylan B. Tierney, Molly F. Franke, Mercedes C. Becerra, Félix A. Alcántara Virú, César A. Bonilla, Epifanio Sánchez, Dalia Guerra, Maribel Muñoz, Karim Llaro, Eda Palacios, Lorena Mestanza, Rocío M. Hurtado, Jennifer J. Furin, Sonya Shin, Carole D. Mitnick

**Affiliations:** 1 Division of Global Health Equity, Brigham and Women's Hospital, Boston, Massachusetts, United States of America; 2 Partners In Health, Boston, Massachusetts, United States of America; Socios En Salud Sucursal Peru, Lima, Peru; 3 Department of Global Health and Social Medicine, Harvard Medical School, Boston, Massachusetts, United States of America; 4 National Tuberculosis Strategy, Ministry of Health, Lima, Peru; 5 Hospital Nacional Sergio E. Bernales, Lima, Peru; 6 Asociacion Visionarios, Peru, Lima, Peru; 7 Division of Infectious Diseases, Massachusetts General Hospital, Boston, Massachusetts, United States of America; 8 Global Health Committee, Boston, Massachusetts, United States of America; 9 Tuberculosis Research Unit, Case Western Reserve University, Cleveland, Ohio, United States of America; McGill University, Canada

## Abstract

Sputum cultures are an important tool in monitoring the response to tuberculosis treatment, especially in multidrug-resistant tuberculosis. There has, however, been little study of the effect of treatment regimen composition on culture conversion. Well-designed clinical trials of new anti-tuberculosis drugs require this information to establish optimized background regimens for comparison. We conducted a retrospective cohort study to assess whether the use of an aggressive multidrug-resistant tuberculosis regimen was associated with more rapid sputum culture conversion. We conducted Cox proportional-hazards analyses to examine the relationship between receipt of an aggressive regimen for the 14 prior consecutive days and sputum culture conversion. Sputum culture conversion was achieved in 519 (87.7%) of the 592 patients studied. Among patients who had sputum culture conversion, the median time to conversion was 59 days (IQR: 31–92). In 480 patients (92.5% of those with conversion), conversion occurred within the first six months of treatment. Exposure to an aggressive regimen was independently associated with sputum culture conversion during the first six months of treatment (HR: 1.36; 95% CI: 1.10, 1.69). Infection with human immunodeficiency virus (HR 3.36; 95% CI: 1.47, 7.72) and receiving less exposure to tuberculosis treatment prior to the individualized multidrug-resistant tuberculosis regimen (HR: 1.58; 95% CI: 1.28, 1.95) were also independently positively associated with conversion. Tachycardia (HR: 0.77; 95% CI: 0.61, 0.98) and respiratory difficulty (HR: 0.78; 95% CI: 0.62, 0.97) were independently associated with a lower rate of conversion. This study is the first demonstrating that the composition of the multidrug-resistant tuberculosis treatment regimen influences the time to culture conversion. These results support the use of an aggressive regimen as the optimized background regimen in trials of new anti-TB drugs.

## Introduction

Sputum cultures are an important tool in monitoring the response to tuberculosis (TB) treatment, especially in multidrug-resistant TB (MDR-TB). Cultures are usually obtained on a monthly basis while under therapy, if resources are available [1]. Sputum culture conversion—the transition in sputum culture results from a positive sample growing *Mycobacterium tuberculosis* to two consecutive negative cultures separated by at least 30 days —is a key clinical milestone signifying that the patient is responding to therapy [2], [3].

It is known that culture conversion can be delayed in the treatment of MDR-TB compared to drug-susceptible disease [4]. Risk factors for delayed culture conversion during MDR-TB therapy include markers of advanced pulmonary disease, specifically smear positivity [5], [6] and the presence of cavitary lung lesions [7]. Culture conversion has also been shown to occur more slowly in patients with resistance to second line anti-TB drugs [8], [9]. More recently, co-morbidities like low body weight [10] and smoking [11] have been linked to delayed conversion.

There has, however, been little study of the effect of treatment regimen composition on culture conversion. In addition to the potential benefits for patient care, such information is also critical to the design of MDR-TB treatment trials [12]. Pivotal phase II studies of the new anti-TB drugs bedaquiline and delamanid used designs that compared sputum culture conversion in patients who received the standard-of-care—also known as the optimized background regimen—and placebo to patients who received the optimized background regimen plus the investigational agent [13], [14]. The percentage of participants in the placebo arm whose sputum culture converted at two months was different between the two studies, but reasons for this difference have not been clearly discerned. Differing composition of the optimized background regimen is one possible explanation.

We conducted a retrospective cohort study to assess whether treatment regimen composition, particularly whether an aggressive regimen containing five likely effective drugs including a fluoroquinolone and an injectable agent during the intensive phase was associated with accelerated sputum culture conversion.

## Methods

### Study population

The study population comprised patients starting treatment for MDR-TB in Lima, Peru, during the period 1 February 1999 to 31 July 2002. Patients were included in the analysis if the regimen they received during the study period was their first regimen individualized to drug susceptibility test results. Patients were excluded if they did not have a positive culture at the start of treatment or if they had a positive culture followed by a negative culture prior to the start of treatment. Patients were also excluded if data were not available about the composition of their individualized MDR-TB treatment regimen.

The approach to drug susceptibility testing (DST), treatment and monitoring in this cohort has been previously described [15]. All DST was performed at or under the guidance of a supranational reference laboratory. Isolates were routinely tested for isoniazid, rifampin, pyrazinamide, ethambutol, and streptomycin susceptibility. Second-line DST (amikacin, capreomycin, cycloserine, ethionamide, kanamycin, *para*-aminosalacylic acid, ciprofloxacin or ofloxacin and either gatifloxacin, levofloxacin or moxifloxacin) was also performed on a majority of the patients. These DST results as well as any prior anti-TB drug exposure were taken into account when formulating the treatment regimen [16].

Patients were closely monitored during treatment. Directly observed therapy was provided by local community health workers and program nurses. Adverse events were managed in consultation with supervising physicians from the Peruvian National TB Program. Additionally, patients were given nutritional, psychological and financial supports while on treatment.

### Primary exposure and covariates

The primary exposure was receipt of an aggressive treatment regimen, examined as a time-varying variable. An aggressive regimen is an MDR-TB treatment composed of at least five likely effective anti-TB agents, including a fluoroquinolone, and in the intensive phase, an injectable agent [15]. Injectable agents included streptomycin, kanamycin, amikacin and capreomycin. Fluoroquinolones included ciprofloxacin, ofloxacin, levofloxacin or moxifloxacin. Agents were classified as likely effective if the baseline phenotypic DST showed no evidence of resistance to that agent or, if DST was not available, if the patient had less than one month of exposure to the drug prior to starting the MDR-TB regimen.

**Figure 1:**
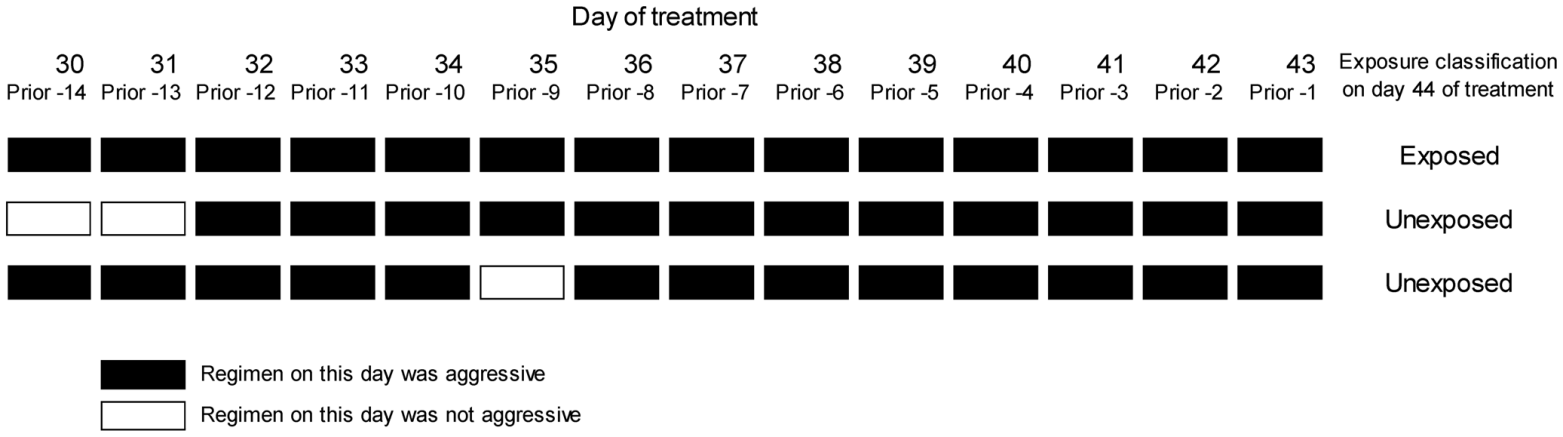
Illustration of daily exposure status defined by receipt of an aggressive regimen in the 14 days prior (example of three patients assessed at day 44 of treatment).

Exposure was defined daily, based on receipt of the aggressive regimen for the prior 14 days: if the treatment met the definition of aggressive on all of the previous 14 days, that patient-day was considered exposed. (Figure 1 illustrates an example of exposure classification.) Exposure status was recalculated—and could change—on each day that the patient was on treatment through the entire treatment period until culture conversion or censoring. We chose a 14-day exposure interval reasoning that it is a plausible period in which to expect conversion [17]. Exposure intervals shorter than 14 days were rejected given concerns that second-line anti-TB drugs are generally considered less efficacious compared to first-line therapy. Covariates included demographic variables, prior TB treatment, date of treatment, indicators of disease severity, and comorbidities.

### Outcome definition

The primary outcome was time to sputum culture conversion. Sputum culture conversion was defined as two consecutive negative cultures separated by at least 30 days. The date of sputum culture conversion was the date of the first of two consecutive sputum cultures to be recorded as negative in a patient who had culture-converted [2].

### Statistical analysis

We conducted Cox proportional-hazards analyses, stratified by day of treatment using the Anderson-Gill formulation [18], [19], to examine the relationship between receipt of an aggressive regimen for 14 prior consecutive days and time to sputum culture conversion. Individuals without culture conversion were censored on the earlier of their last culture date or treatment outcome date. To account for possible informative censoring by death, we adjusted for variables that had been associated with mortality in a previous analysis of this cohort: age, female gender, HIV infection, at least one comorbidity other than HIV, the number of resistant agents at baseline, having received less than two previous regimens or a standardized MDR-TB regimen, tachycardia, low BMI, and extrapulmonary TB [15]. Additionally, we adjusted for all other covariates shown in Table 1 and removed them using a manual backward selection approach if they did not predict the outcome at a p- value ≤0.20 and if removal did not alter the hazard ratio for the aggressive regimen variable by >10%. For multivariable analysis, missing values were multiply imputed using Markov Chain Monte Carlo methods to complete the dataset. We examined potential violations of the proportional hazards assumption by including an interaction term between receipt of an aggressive regimen and time since the start date of the MDR-TB regimen (1–6, 7–12, >12 months). This allowed us to assess the presence of effect modification by time.

**Table 1:** Distribution of covariates at initiation of the individualized MDR-TB regimen.

COVARIATE	N	Patients with specified characteristics
		N (%) or Median (IQR)
PRIOR TREATMENT		
Received ≤2 previous regimens without prior standardized regimen for MDR-TB	592	160 (27.0)
DEMOGRAPHICS		
Female	592	227 (38.3)
Age	592	28.7 (23.0–37.8)
Enrolled in Northern Lima	592	252 (42.6)
Enrolled prior to March 1, 2001*	592	135 (22.8)
INDICATORS OF SEVERITY		
Bilateral, cavitary findings	568	314 (55.3)
Low BMI or malnutrition‡	508	190 (37.4)
Low hematocrit€	521	249 (47.8)
Tachycardia	578	169 (29.2)
Respiratory difficulty¥	563	404 (71.8)
Extrapulmonary TB	592	46 (7.8)
Number of resistant agents	592	5 (4–7)
Lab-confirmed XDR-TB£	577	45 (7.8)
Prior resective surgery	579	16 (2.8)
COMORBITIES		
Patients with at least one comorbidity§	573	206 (36.0)
HIV infection	583	7 (1.2)

*Patients enrolled prior to March 1, 2001 were more likely to have received the standard Category II retreatment regimen after failure of Category I than patients enrolled after this date, when national policy changed.

‡ <18.5 in women, <20 in men; or malnutrition established clinically.

€ ≤30% in women, ≤36% in men; when missing, also used hemoglobin ≤10 in women and ≤12 in men.

¥ Dyspnea; resting heart rate >26/minute.

£ Isolate resistant to at least isoniazid, rifampin, fluoroquinolone, and injectable (kanamycin, capreomycin or amikacin).

§ This includes the following comorbidities: cardiovascular disease (12), diabetes (15), hepatitis or cirrhosis (7), epilepsy/seizures (10), renal insufficiency (6), psychiatric disorder (101), ever smoked (59), ever used/abused alcohol or other substance (49).

### Data collection and availability

Data were collected and recorded in a web-based electronic medical record during treatment. A standardized chart abstraction was conducted to complete the dataset. Data has been deposited in a public repository at the Harvard Dataverse (doi: 10.7910/DVN/27020).

### Ethics statement

The parent study was first approved by the Institutional Review Board at Harvard Medical School and by the Ministry of Health of Peru in 2000. At the time of the reviews, the informed consent form used routinely by the Peruvian National Tuberculosis Program for MDR-TB patients was deemed to be sufficient. As a result, no waiver was obtained. The authors had no access to identifying information during the current study or previous parent study. Data was anonymized prior to analysis.

## Results

We found 673 patients available for analysis. Eighty-one patients were excluded: two because they had already received an individualized regimen for MDR-TB, two for incomplete treatment regimen data and 77 because they did not have a positive culture prior to the initiation of the individualized MDR-TB regimen or because a more recent negative culture followed the positive culture that preceded the start of the individualized MDR-TB regimen. A total of 592 patients were analyzed.

Baseline characteristics of the study cohort are presented in Table 1. Two hundred and twenty seven (38.3%) patients were female. The median age was 28.7 (inter-quartile range [IQR]: 23.0–37.8) years. Drug resistance was common in the cohort, with cultures resistant to a median of five (IQR 4–7) drugs. There were 45 (7.8%) patients who had extensively drug-resistant TB. Advanced pulmonary disease was also common: 404 (71.8%) patients had respiratory difficulty at treatment initiation and 314 (55.3%) patients had bilateral or cavitary disease on chest radiograph. Co-morbidities were present in 206 (36.0%) patients. Only seven (1.2%) patients, however, had HIV co-infection.

Sputum culture conversion was achieved in 519 patients (87.7%). Among patients who had sputum culture conversion, the median time to sputum conversion was 59 days (IQR: 31–92). In 480 patients (92.5% of those with conversion), conversion occurred within the first six months of treatment. The rate of conversion was highest in months 0–6, after which time the rate was notably lower (Table 2).

**Table 2:** Rates of sputum culture conversion in patients receiving individualized MDR-TB regimens, by treatment semester.

Time since start of MDR-TB regimen	Conversions	Total person-time (months)	Rate per 100 person months [95% CI]
0–6 months	480	1514.6	31.7 [29.0, 34.6]
7–12 months	26	258.0	10.1 [6.7, 14.5]
>12 months	13	265.9	4.9 [2.7, 8.1]

In univariate analysis, exposure to an aggressive regimen was significantly associated with sputum culture conversion (hazard ratio [HR] 1.40; 95% confidence interval [CI] 1.17, 1.67).

**Figure 2:**
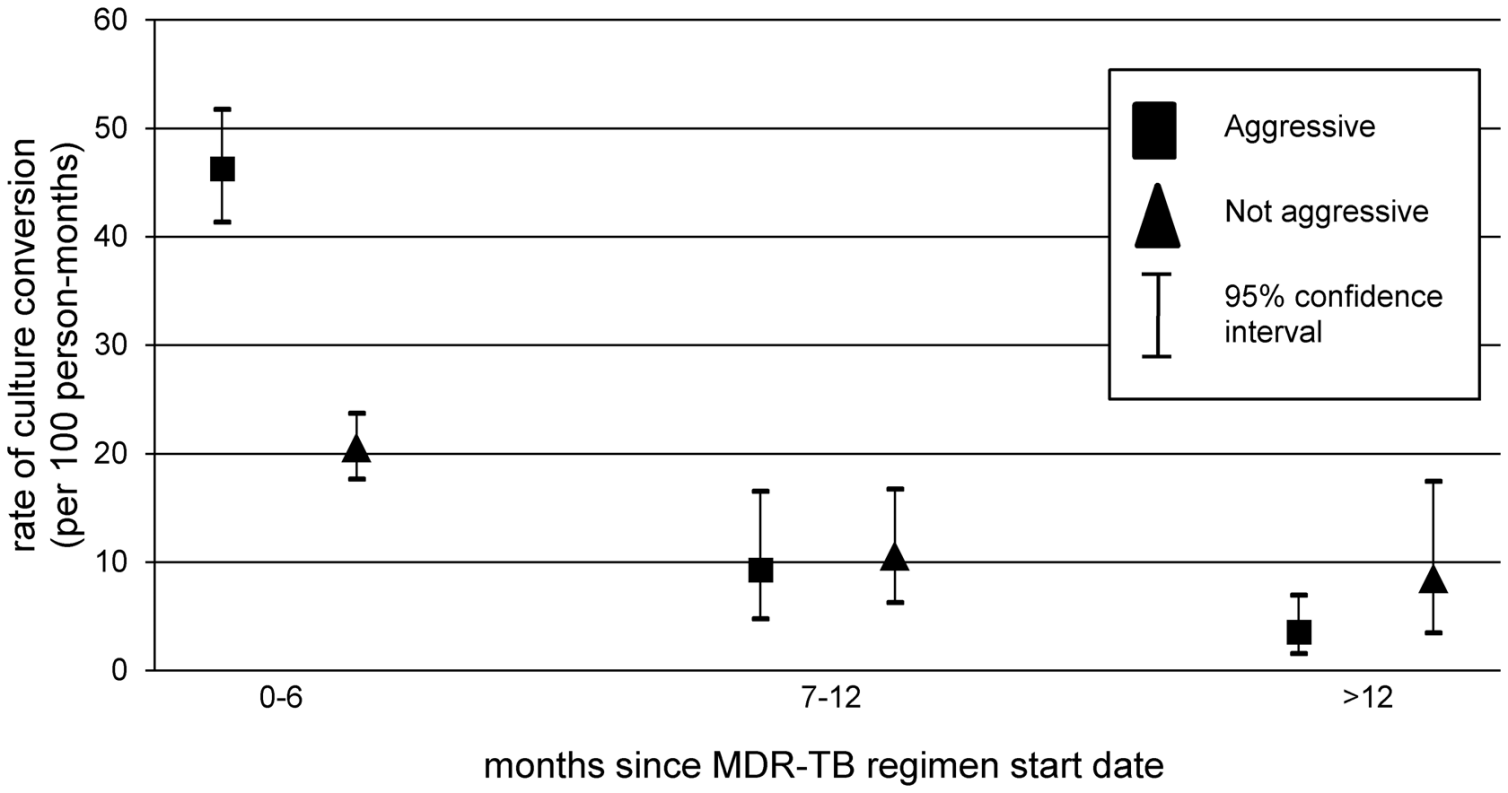
Aggressive regimens are associated with unadjusted rates of culture conversion in the first six months of treatment.

In our assessment of the proportional hazards assumption, we observed effect modification by time since MDR-TB treatment initiation. Specifically, we noted that the association between an aggressive regimen and culture conversion varied by time since treatment initiation (0–6 months, 6–12 months, >12 months) (Figure 2). In multivariable analysis, exposure to an aggressive regimen was independently associated with sputum culture conversion during the first six-months of treatment (HR: 1.36; 95% CI: 1.10, 1.69), but not during months 6–12 of treatment (HR: 0.95; 95% CI: 0.42, 2.15) or after 12 months of treatment, (HR: 0.39; 95% CI: 0.12, 1.24; likelihood ratio p-value for interaction with time on treatment: 0.09) (Table 3).

**Table 3:** Multivariable proportional-hazards analysis of aggressive regimen and sputum culture conversion.

Variable	Hazard ratio, multivariable analysis	95% CI, multivariable analysis	P value
Aggressive regimen for 14 prior consecutive days			
1–6 of treatment	1.36	1.10, 1.69	0.005
7–12 of treatment	0.95	0.42, 2.15	0.93
after 12 months of treatment	0.39	0.12, 1.24	0.11
Female	0.90	0.75, 1.09	0.27
Age	1.00	0.99, 1.01	0.76
At least one comorbidity, other than HIV	0.85	0.70, 1.03	0.10
HIV	3.36	1.47, 7.72	0.004
Number of resistant agents	0.94	0.88, 1.00	0.07
Tachycardia	0.77	0.61, 0.98	0.03
Low BMI or malnutrition	0.87	0.70, 1.08	0.20
Extrapulmonary TB	0.94	0.65, 1.37	0.76
Received ≤ previous regimens without prior standardized regimen for MDR-TB	1.58	1.28, 1.95	<0.0001
Low hematocrit	0.85	0.69, 1.03	0.10
Respiratory difficulty	0.78	0.62, 0.97	0.02
Treatment period	0.83	0.67, 1.03	0.09

All subsequent results are from the model that accounts for the effect modification. Infection with HIV (HR 3.36; 95% CI: 1.47, 7.72), and receiving less exposure to TB treatment prior to the individualized MDR-TB regimen (HR: 1.58; 95% CI: 01.28, 1.95) were also independently positively associated with conversion. Tachycardia (HR: 0.77; 95% CI: 0.61, 0.98) and respiratory difficulty (HR: 0.78; 95% CI: 0.62, 0.97) were independently associated with a lower rate of conversion.

## Discussion

We found that patients treated for MDR-TB had higher rates of sputum culture conversion if they received an aggressive regimen for MDR-TB for the 14 preceding consecutive days compared to those who did not. To our knowledge, this is the first report to demonstrate an association between regimen composition and time to culture conversion in the treatment of MDR-TB.

Infection with HIV was also associated with more rapid sputum conversion. This finding may be related to the pauci-bacillary state of HIV and TB coinfection, with lower amounts of TB bacilli per milliliter of sputum in patients with HIV infection [20]. Sputum culture conversion was also more likely if the patient had not received more than two previous regimens or a standardized MDR-TB regimen, highlighting the importance of prompt receipt of appropriate treatment for MDR-TB.

In contrast, indicators of advanced pulmonary disease like respiratory difficulty and tachycardia were associated with lower rates of conversion, possibly because of higher bacterial burdens in those patients. Alternatively, patients with advanced disease may also have damaged lung parenchyma, thereby limiting the penetration of drug into the most diseased tissue. It has been previously shown that cavitary disease also influences rates of conversion [7], [21].

We found that culture conversion rates were higher in the first six months of treatment compared to later. High rates of culture conversion within the first six months of effective MDR-TB treatment have also been shown in the past [22]. One possible explanation for lower conversion rates later in treatment is that patients who did not convert in the first six months may have been sicker and less likely to experience conversion on any regimen. Our observation that indicators of severe disease were associated with delayed conversion supports this explanation. We further observed that an aggressive regimen did not confer the same benefit later in therapy. The apparent waning of the aggressive regimen's effect on culture conversion over time during treatment may be related to the gradual depletion of individuals who are likely to benefit from an aggressive regimen.

From these findings, we posit that there is a majority of patients for whom the aggressive regimen will shorten the time to culture conversion. Because the transition from the injectable-based intensive phase of MDR-TB treatment to the continuation phase depends on culture conversion, the aggressive regimen may carry a potential benefit in shortening the duration of the intensive phase, thereby limiting the toxicity and programmatic costs associated with this part of treatment.

There are, however, other patients who may not convert in the first six months of treatment using current MDR-TB regimens. Further characterization of these patients with delayed conversion is needed to determine potential causes of persistent culture positivity. For patients who do not convert in the first six months, additional strategies/interventions may be needed to effect conversion. Some of these additional interventions include enhanced adherence support, aggressive management of adverse events, and consideration of resective surgery.

Interest in using culture conversion as a surrogate clinical endpoint is mounting because of its potential to shorten the time under observation in clinical trials of new anti-TB drugs [23], [24]. The favored approach to investigating activity of new drugs against MDR-TB has been to add either placebo or a new agent to an optimized background regimen and compare rates of culture conversion [25]. Two recent studies revealed the benefit conferred by the addition of investigational agents using this method [13], [14]. There was, however, considerable variability in the composition of the optimized background regimens: one was standardized, comprising kanamycin, ofloxacin, prothionamide, terizidone and pyrazinamide, while the other was individualized in accordance with WHO recommendations. The frequency of and time to culture conversion among participants in the control arms of the two studies was also very different; in the former, only 9% of patients converted at two months, while in the latter, 30% converted. Our results reveal that regimen composition can be a chief source of such variability in early treatment response, independent of other sources, and raises the possibility that a portion of the difference in conversion in the aforementioned studies may have been due to the composition of the background regimen. The implication of such a finding is that the benefit of a new drug may be overestimated if it is added to a weak background regimen.

In our study, there exists the possibility of misclassification of the exposure by defining exposure as receipt of 14 consecutive days of the aggressive regimen. Patients who had received an aggressive regimen during most but not all of the 14-day window may still have benefited, but they were classified as unexposed. We would expect that this misclassification would attenuate the observed hazard ratio for the aggressive regimen variable.

Although this study was conducted among patients with significant prior treatment exposure, including to second-line drugs, our conclusion—aggressive regimens accelerate conversion—should be generalizable to patient populations with differing patterns of prior exposure and other baseline characteristics. The key to formulating these aggressive regimens is integrating accurate information about prior treatment exposure with cautious interpretation of DST when deciding which agents are likely to be effective against the infecting strain. Although the magnitude of the benefit may vary across populations, the application of these principles is likely to improve outcomes independently of the distribution of baseline characteristics.

We have now demonstrated that culture conversion in MDR-TB can be influenced by the component anti-TB drugs in a treatment regimen. Specifically, we have shown that an aggressive regimen for MDR-TB is significantly associated with accelerated culture conversion, which adds to a growing body of knowledge on the benefits of this regimen including reduced risk of death [15], failure [26], and relapse [27]. This cumulative evidence suggests that aggressive regimens with at least five likely effective drugs including an injectable and fluoroquinolone should be considered the standard of care in the treatment of patients with MDR-TB. Further, optimized background regimens in treatment trials should be constructed to meet this new standard of care. In this way, the comparison group will receive a treatment known to achieve the best possible outcome, including the most rapid rate of culture conversion, thereby establishing a benchmark upon which experimental regimens must seek to improve.
